# Removal of Six Inches of Small Intestine

**Published:** 1897-01

**Authors:** Hunter P. Cooper

**Affiliations:** Atlanta, Ga.; Professor of Anatomy and Clinical Surgery, Atlanta Medical College; Surgeon to the Grady Hospital


### REMOVAL OF SIX INCHES OF SMALL INTESTINE. UNION OF THE DIVIDED ENDS BY MEANS OF THE MURPHY BUTTON.

By HUNTER P. COOPER, M.D.,

Professor of Anatomy and Clinical Surgery, Atlanta Medical College; Surgeon to the Grady Hospital.

Mr. M. O., of Cordele, Ga., sent for me September 26th, and gave me the following history: He was operated on in April last for appendicitis by Dr. John B. Deaver in a hospital in Philadelphia, and he states that the whole appendix was removed. The wound was stitched up apparently without any drainage. Twenty-three days after the operation an abscess formed and discharged through the cicatrix of the wound; this has continued to discharge ever since. Twelve days before coming to Atlanta he began to suffer with constipation, vomiting, and violent abdominal pains. On examining patient I found two discharging sinuses in the cicatrix, and in each of them what appeared to be a silkworm gut suture could be seen. Immediately above the cicatrix was a mass in the abdominal wall half the size of a hen egg and very tender on manipulation. The patient stated positively that there had never been any discharge of fecal matter through these two small openings, nor had any fecal odor been noticed by him in the purulent discharge. I naturally, therefore, thought that I had to deal with an abscess of the abdominal wall caused by the retained sutures.

I etherized him at the Grady Hospital on September 28th, assisted by Dr. Ward, the house surgeon, and the other members of the house staff. I extracted the two sutures, which were found to be of silkworm gut. I then carefully cut through the bridge of tissue between the two sinuses in order to give a free vent to the discharge. As soon as the blood and discharge had been washed away

I found, to my horror, that I was in the cavity of the small intestine. A close examination then revealed a large opening in the small intestine at a point opposite its mesentery. This opening was over an inch long, and extended transversely nearly half the diameter of the bowel. Its edges were ragged and ulcerated, and firmly adherent to the cicatrix. My knife had made no part of the opening. The edges everywhere were thick and inflamed, and it was apparent he had suffered no fecal extravasation, because of the very minute size of the openings leading from the cavity of the bowel to the skin. Of course it was evident that the operation had at once assumed much graver proportions than were apparent at first. There was nothing to be done but to endeavor to effect a closure of this opening, and its size both longitudinally and transversely precluded the idea of anything else than a resection of this part of the bowel. I therefore dissected the edges of the opening away from the abdominal wall, and found myself in a mass of inflammatory adhesions very dense and vascular, in which a coil of the small intestine was matted in the omentum, mesentery and adjacent coils of intestines.

After a most tedious dissection the part of the intestine involved was released and drawn up into the wound. It was then found that this whole section of the intestine was inflamed and thickened; its mesentery thick and indurated, and the peritoneum stripped off it over quite a large area, wherever it had been adherent to other structures. I then tied off the mesentery for about six inches along the border attached to the intestine, compressing the intestine above and below, and excised about six inches of it. The Murphy button was then introduced into each end of the cut intestine, and an end-to-end approximation made. The abdomen was then thoroughly washed out with a large quantity of sterilized hot water, and a large iodoform gauze wick introduced into the abdominal cavity with its lower end reaching the part of the intestine where the button was inserted. The wound was then stitched up, leaving an opening in its middle through which the iodoform gauze was brought for drainage. On account of the numerous adhesions and the large number of ligatures that were necessary to control the hemorrhage, the operation consumed three hours. The patient stood it fairly well, and came out from the effects of the ether without any nausea or vomiting. When the operation was about half concluded Dr. Elkin came into the room and kindly remained and lent me valuable assistance.

September 29th. Patient was very restless for a number of hours after recovering from the ether, and by my direction Dr. Ward, the house physician, gave him several hypodermics of one-eighth of a grain of morphine. He passed a fairly comfortable night, and this morning his temperature was $98\frac{2}{5}$, pulse 94. He had no vomiting, there was no distension of the abdomen, and he had been able to retain milk every three hours in small quantities.

Five P.M. Temperature $99\frac{1}{5}$, pulse 100. Patient has not vomited at all to-day, and has taken milk every three hours; complains a good deal of abdominal pain, but there is no distension of the bowels.

September 30th, 9 A.M. Temperture $98\frac{4}{5}$, pulse 94. Patient had a pretty comfortable night, although he did not sleep much. Vomited some curdled milk once in the night. No distension of the abdomen and no pain. He took last night three grains of calomel at 8 P.M., and a small dose of salts this morning.

Six P.M. Has been nauseated all day. At 1:45 vomited over a pint of green fluid; temperature 98, pulse 98. Discontinued the salts in the forenoon on account of his nausea. Ordered one-quarter of a grain morphine hypodermically at 8 P.M.

October 1st, 7 A.M. Temperature $98\frac{3}{5}$, pulse 84. Slept quietly large part of the night. Had no vomiting, although he complains of nausea all the time. Has retained nourishment twice since midnight. No distension of the abdomen and no pain. Bowels have not moved, although he made an effort after using an oil enema.

Six P.M. Temperature $99\frac{3}{5}$, pulse 78. Has taken nourishment to-day every three hours, vomited once; bowels have not moved. He is quite comfortable.

October 2d, 9 A.M. Temperature $99\frac{1}{5}$, pulse 80. Had a very comfortable night, sleeping most of the time. Has not vomited. This morning early he had a large, free, natural action. Wound was dressed this morning, and the gauze drainage removed. There is no distension of the abdomen, and his general condition seems much improved.

October 5th. Patient has done very well ever since last note. This morning on removing the dressing, there was a distinct fecal odor in the discharge. Late this evening fecal matter came through freely, soiling the dressings and bed.

October 10th. Since last note patient's general condition has been excellent, but there has been very free discharge of fecal matter in the dressings.

October 14th. During the past four days the fecal discharge has been rapidly diminishing, and is now very slight. He passed the button last night.

October 15th. During the past twenty-four hours there has practically been no discharge of fecal matter whatever from the wound.

I should remark that the whole wound united by first intention, except at the point where the gauze drainage came out, and it was at this point that the fecal matter made its discharge.

October 24th. Since last note there has been no fecal discharge whatever from the wound; granulations now entirely fill the wound, and there is a small granulating surface not larger than the little finger nail which has still to heal. Patient discharged from the hospital to-day.

He returned to his home about November 1st, at which time there was simply a little granulating spot, and he had gained flesh and strength very rapidly.

There are several remarkable features about this case. In the first place, it is very remarkable how a person with such a large opening in the small intestine could remain so free from fecal discharge externally. Second, what is to me a still more remarkable fact, is the rapidity with which the fecal fistula which followed the operation healed. I would also like to state that the insertion of the Murphy button renders end-to-end anastomosis of the intestine a matter of great simplicity.

